# Sexual motivations during the menopausal transition among Iranian women: a qualitative inquiry

**DOI:** 10.1186/s12905-018-0684-z

**Published:** 2018-11-23

**Authors:** Zeinab Javadivala, Effat Merghati-Khoei, Carol Underwood, Mojgan Mirghafourvand, Hamid Allahverdipour

**Affiliations:** 1 Department of Health Education & Promotion, Tabriz University of Medical Sciences, Tabriz, Iran; 2 Iranian National Center for Addiction Studies (INCAS), Tehran University of Medical Sciences, Tehran, Iran; 3 Brain and Spinal Cord Injury Research Center (BASIR), Tehran University of Medical Sciences, Tehran, Iran; 4 Department of Health, Behavior, and Society Bloomberg School of Public Health, Johns Hopkins University, Baltimore, USA; 5 Department of Midwifery, Tabriz University of Medical Sciences, Tabriz, Iran; 6 Research center for psychiatry and behavioral sciences, Tabriz University of Medical Sciences, Tabriz, Iran

**Keywords:** Sexual motivations, Menopause, Iranian women, Sociocultural scripts

## Abstract

**Background:**

Women’s sexuality may be adversely affected during the menopausal transition. This exploratory qualitative study was conducted to explore how women assign meaning to and process sexual motivation during the menopausal transition.

**Methods:**

We purposefully approached 22 married women ages 44–59 (52.81 ± 3.6 years) in urban health care centers and workplaces in Tabriz city, located in northwest Iran. Individual face-to-face interviews were performed at a place and time convenient to the women. All interviews were audio recorded and then transcribed to create verbatim written accounts. Inspiring Graneheim and Lundman approach, we employed conventional content analysis to derive coding categories directly from our row data.

**Findings:**

Four main themes emerged from data analysis: “Diminished sexual capacity” (effect of menopause, Illnesses associated with mid-life, desire discrepancy); “intimate coupling” (lack of physical and/or emotional intimacy, couple communication and romance); “sociocultural scripts” (sexual script, parental responsibilities); and “sense of youthfulness” (having an active and happy life, maintaining physically attractiveness).

**Conclusion:**

The qualitative findings suggest that providing sexual health education and counseling, to encourage critical discussions regarding current sociocultural scripts and to create an environment that would enable men and women alike to adopt a healthy and happy lifestyle for eliminating barriers and preserving and enhancing motivational factors associated with sexuality.

## Background

Sexuality, culture and gender are historically interwoven [1, 2]. This paper focuses on the meanings of sexuality for Iranian women who are experiencing menopause. The intention here is neither to represent biological perspectives nor to speak for all Iranian women. Rather, the goal is to gain insight into the menopausal experiences of Iranian women from a range of backgrounds. In many societies, conversations about sex and related themes are avoided by women and are often considered taboo [3–5]; Iran is not an exception. As in other settings, sociocultural factors strongly influence most aspects of sexual life [6]. Cultural issues, social contexts, values and norms may limit sexual expression and, consequently, suppress sexual motivation during the menopausal transition [3]. In Iran, religious culture forms the basis for most Iranians’ understandings of sexuality [1]. This paper reports findings from interviews with study participants about sexual motivation during the menopausal transition.

Many, even most, women experience difficulties in their sexual lives during menopausal transition [7]. Female sexual dysfunction (FSD) is common among women; it is estimated that 40–50% of peri- and postmenopausal women suffer from FSD [8]. Merghati-Khoei, etal. (2014) found that sexual desire was decreased or absent in 94.5% of the 200 postmenopausal women who participated in their study [9]. Another cross-sectional study in Iran indicated that 70% of women had reported a decrease in sexual activities after menopause [7]. Impaired sexual motivation has been identified as one of the main domains in the formation of women’s sexual problems [10].

Female sexuality is a multifaceted, complex phenomenon influenced by the interplay of biological, psychological and socio-cultural factors [8, 11]. Moreover, partner-related factors such as partner’s health status and age as well as the quality and duration of a given relationship influence women’s sexuality [12]. There is now consistent evidence that the effects of these factors on women’s sexual experiences are at least as important as the biological aspects of menopause [12, 13].Moreover, these problems can affect multiple aspects of menopausal women’s sexual lives, including sexual capacity, excitement, motivation and function [14].

Sexual motivation has been described as what encourages or prompts sexual interaction [15]. Motivations for and against sex are salient predictors of whether and when an individual will engage in or abstain from sex [16]. Significant evidences point to relational intimacy, including emotional closeness, bonding and commitment, as important motives for engaging in sexual activity [17, 18].Sexual challenges may prevent couples from generating and sustaining the motivation required to be sexuality active [17]. Yet, one study found that, from the perspective of most women (76%), a satisfactory sex life is essential to maintaining a relationship and more than half of women (57%) found life without a sexual relationship disagreeable [8].

To assess sexual well-being and provide treatment or education for women’s sexuality, it is necessary to understand the factors associated with sexual motivation in each context, culture and country. Iranian women’s sexual relationships and encounters often take place in the context of the intimacy and commitment of marriage [15]. In Iran, there is often a strong emotional relation between wives and husbands; a woman’s sexual enjoyment is typically focused on relations with her husband. Iranian women report that feeling loved by their husband is their main motive for sex [19]. Other factors reported included having sex to meet the couple’s physical needs and as an aspect of taking control of life [19]. Yet, talking about sex is often considered taboo; modesty and shyness related to this topic prevent women – in particular – from conversing about sex [4, 7]. Iranian women describe satisfaction with sex when it takes place in a private and safe context, and within the parameters accepted by the society and religion [19].

Our search of the literature found that studies about Iranian women’s sexuality during the menopausal transition are very limited [20], largely due to the unspoken nature of sexuality in Iranian culture. Most studies in this area have been quantitative and informed by a positivist paradigm. To better understand how women assign meaning to and process sexual motivation during the menopausal transition, a more interpretive, phenomenological approach that relies on idiographic methods can provide valuable insights. This exploratory qualitative study was conducted to help fill that gap.

## Methods

We began by asking several women with knowledge of women’s sexual health concerns how we could best access potential participants. Based on these initial inquires, we interviewed five women face-to-face. These key informants then led us to urban health care centers and workplaces in Tabriz city, located in northwest Iran. Approaching women through the centers facilitated maximizing variation sampling in terms of age, residence, socioeconomic status, educational attainment and work experience. Despite the assumption that speaking about sexuality is difficult for Iranian women, study participants easily opened up with respect to their menopausal sexual lives and shared their understandings and experiences with the researchers. In total, 22 married women ages 44–59 (52.81 ± 3.6 years) who were living with their husbands at the time of the interview participated in study. Participants were Iranian, spoke Azeri and had no history of premature menopause, hysterectomy or oophorectomy or history of hormonal replacement therapy. Participants who expressed their willingness to participate in the study were consented. More details about the participants are presented in (Table 1). Individual face-to-face interviews were performed at a place and time convenient to the women. For comfort and to protect confidentiality, no one other than the participants and interviewer were present during the interviews; thus, no participant refused to participate or dropped out.

**Table 1:** Participants’ demographic data

Demographic data	Number	Percent
Age(year)		
40–44	2	9.1
45–49	5	22.7
50–54	8	36.3
55–60	7	31.9
Education		
Primary	7	31.8
Secondary	11	50
University	4	18.2
Occupation		
Housewives	17	77.3
Employed	5	22.7
Menopause transitional stage ^a^		
Perimenopause ^b^	11	50
Post menopause ^c^	11	50

^a^As defined by Stages of Reproductive Aging Workshop –STRAW

^b^Perimenopause is characterized by a woman having regular menstrual cycles with interval changes of 7 days or more and/or two or more skipped menstrual cycles and at least one inter-menstrual interval of 60 days or more

^c^Post-menopause is the stage reached when 12 months has passed since the final menstrual period

Menopausal women: Both perimenopausal and post-menopausal women

### Data collection

Each consented participant was interviewed at least once; interviews lasted 40–100 min. Interviews were conducted by two of the female authors (ZJ and EMK) who specialize (PhD) in health promotion and sexology. If, during the interview, an ambiguous expression or unclear point arose, additional exploratory questions were asked using the mirroring technique, where interviewer reflect the participant’s answer back to her in question form. Furthermore, when unclear points were found during the initial analysis, an additional interview with the participant in question was conducted. The goals of the study were explained to participants and consented to take part in the study and to have their interviews recorded. Privacy, the right to withdraw from the study and other ethical commitments were explained. Because of face to face meeting, except than interviewer nobody of research team knew the name of participants. Interviews started with general questions about feelings and perceptions associated with menopause. The interviewers continued by asking participants questions related to their perceptions about sexual life during the peri-menopausal period, any recent changes in sexual motivation they had experienced, and enabling or inhibiting factors affecting sexual motivation during their menopause transition. Probes were tailored to participants’ responses. All interviews were audio recorded and then transcribed to create verbatim written accounts. Field notes were taken immediately after each interview.

#### Data analysis

We employed conventional content analysis to derive coding categories directly from our row data [21]. Inspiring Graneheim and Lundman approach, whole transcribed texts were broken down into meaning units. Meaning units are simply small pieces that are created from breaking down the main text. Then, each meaning unit was abstracted to a “condensed meaning unit”. Based on these units, codes were extracted. Then the codes were classified into categories based on their similarities and the relationship between them was determined [22]. Findings generated from content analysis are based on the unique views of the participants and are rooted in textual data. The first step of the analysis was to identify the units of meaning that emerged from the statements. Codes were then generated inductively, and the extracted codes were identified as categories based on differences and similarities. Data analysis continued until data saturation was achieved, or at the point that no new theme or idea emerged. Related meaning units were identified and coded. Codes were compared based on similarities and differences and classified into categories and subcategories, reflecting the manifest content of the text. Examples of the content analysis, encoding, subthemes, and main themes are shown in Table 2. Next, transcripts were converted to rich text format and imported into MAXQ 2007; qualitative software (VERBI Software, Berlin, Germany) was used to organize the data and manage the analysis process.

**Table 2:** An example of analysis process

Meaning unit	Code	Sub themes	Key themes
We have a romantic relationship with each other …it means we love each other a lot, we talk about our feelings easily, We enjoy being with each other and walking, laughing, hugging…	strong emotional intimacy with romantic and friendly relationships	Couple communication and romance	Intimate coupling
My husband always holds me dear. We try to understand each other… by spending time and listening to each other. We mutually respect each other through honorable talking and behavior	effective communication skills		
My husband is my best friend; he helps me a lot. For example we go shopping with each other; he helps me in housekeeping, we usually go on a trip every weekend, he participates in solving our life problems, in brief, we often are with each other. These [factors] motivate me to enjoy being sexually active	true and caring companionship		

#### Data trustworthiness

Credibility was established through prolonged engagement with the participants as well as through member checking, which was carried out in meetings with the respondents to confirm the preliminary findings from the earlier interviews. The research team also met with other experts to conduct peer checking. Moreover, the sampling with maximum variation approach enhanced the credibility of the data as it yielded participants who represented a range of ages, menopausal status, residence, socioeconomic status, educational attainment and work experience. And, finally, re-checking the analytical codes with researchers who had worked with menopausal women in Iran strengthened the confirmability and credibility of the data.

#### Findings

A total of 136 codes, 9 subthemes emerged from data analysis, which were classified into four main themes including: diminished sexual capacity, intimate coupling, sociocultural scripts and sense of youthfulness (Table 3).

**Table 3:** An overview of key themes and sub themes

Key themes	Sub themes
Diminished sexual capacity	-Effects of menopause
	- Illnesses associated with mid-life
	-Desire discrepancy
Intimate coupling	-Lack of emotional/ physical intimacy
	-Couple communication and romance
Socio-cultural scripts	-Sexual scripts
	-Parental responsibilities
Sense of youthfulness	-Having an active and happy life
	-Maintaining physically attractiveness

### Diminished sexual capacity

Sexual capacity as an aspect of human sexual behaviors is defined as “what one can do” [15]. Although sexual desire is only one aspect of sexual capacity, the majority of women used ‘sexual desire, appetite and interest’ to explain their sexual capacity [15]. Women pointed to diminished sexual desire, which they attributed to age-related biological factors that weakened their sexual capacity to engage in sex. This theme was illuminated further through the following three subcategories that women identified as salient during the menopausal transition: effects of menopause, illnesses associated with mid-life and desire discrepancy.

### Effects of menopause

Participants, particularly those experiencing late- or post-menopausal transitions, frequently mentioned “hot flashes” and “vaginal dryness” as factors that had decreased their sexual capacity. Diminished sexual capacity was described as a barrier that suppressed their motivation to engage in sex. Participant 9 said:

*“I cannot stand any hot conditions …even my husband’s body temperature during sex… This is due to hot flashes, so we sleep separately… I think about getting rid of hot flashes instead of having sex*.” (peri-menopause).

Similarly, Participant 10 mentioned:

*“Vaginal dryness and its related pain bother me to the extent that I hate fantasizing about sexual relations or engaging in it*.” (post menopause).

Heavy menses, menses lasting several days longer than usual and menses occurring in close succession were other personal factors related to participants’ flagging sexual motivation, especially those in early- or late-menopausal transition. Participant 8 said:

*“Sometimes my menses occur too close together, even in large volume. After that I feel tired, I like to sleep more, so I lost my willingness to have sex. I often reject my husband’s requests regarding sex relations because I think sex is not important for me anymore.”* (peri-menopause).

### Illnesses associated with mid-life

Respondents who felt vulnerable due to illnesses associated with mid-life reported that their current diseases decreased their sexual motivation. Participant 4 explicitly associated bodily pain with adverse effects on her sexual desire. She felt that menopause had inevitably ruined her sexual motivation and seemed to accept “diminished sexual capacity” as a reality of her sex life:

*“Diseases disturb mental concentration about sexual issues; my bone pain, especially in my feet, has reduced my sexual desire. I think pain has killed my sexual feelings.”* (post menopause).

### Desire discrepancy

Another subcategory of sexual capacity extracted from the analysis of women’s experiences was couple’s desire discrepancy. Some women said that their sexual desire is less than their husbands, indicating that they were unable to respond to their husbands’ sexual needs. This discordance decreased their motivation because of their sense of sexual inadequacy. Participant 3 explained:

*“Our sexual desire and need is not comparable; he is very active, but sex is not very important to me, so I can’t meet his needs. I have a sense of self insufficiency, which has worsened my sexual motivation.”* (perimenopause).

Other women mentioned that their decreased sexual motivation resulted from their husbands’ lack of sexual desire. Participant 9 said:

*“Men’s sexual activity is important in the maintenance of women’s sexual motivation. I think men also experience menopause. My sexual desires used to be important to my husband, but nowadays, he doesn’t react to my sexual actions….so this affecst my motivation, too.”* (perimenopause).

### Intimate coupling

Relationship intimacy is often characterized as closeness, passion and commitment between relationship partners, or the motivation to share one’s private self almost completely with that partner. Couples’ intimate relationship is based in communication and an underlying close emotional attachment. This category was associated with the following sub­categories: lack of emotional/ physical intimacy and couple communication and romance.

### Lack of emotional/ physical intimacy

Emotional intimacy is simply having a very close relationship with one’s spouse and enjoying the feeling of being emotionally close and connected to each other. It allows couples to share personal emotions or feelings with each other. Physical intimacy is sensual proximity or touching. It includes a broad range of physical contacts, including foreplay or non-coital sexual activity, holding hands, hugging, kissing and caressing as well as sexual activity.

There was variability among the participants with respect to their intimate relationships. Some respondents mentioned relational barriers with their husbands that impede their sexual motivation, including the husband’s irritability, criticisms, contemptuous attitudes, lack of companionship and the absence of mutual understanding between spouses. Women who reported living with husbands who are irritable, easily annoyed or prone to anger disrupted both emotional and physical intimacy, as one participant (number 13) noted:

*“He is bad tempered, while we are talking, he is easily annoyed for no reason; he even yells at me. Then, sometimes, doesn’t speak or cuts me off for a short time. Due to his mood, I neither like to approach him nor think about being sexually active anymore.”* (perimenopause).

In a similar vein, Participant 4 asserted:

*“He not only has not appreciated me …but also, from morning to night, he swears at me and my parents. He complains about everyone or everything …..While he wants to have sex, I just think about his contempt….I can’t think about sex… little by little I’ve lost my feelings towards him…. I want to put distance between him and me.” (*post menopause).

Another emotional barrier was lack of companionship and mutual understanding by their spouses, which can dampen any inclination to be intimate with each other. Participant9expressed her experience in this way:

*“My husband doesn’t understand my mood during menopause….he expects me to be like in the past…he not only doesn’t help me in improving my mood, but also, has opted for a solitary life… our relationship is only limited to simple greetings without any physical connections….so I lost my sexuality.”* (perimenopause).

### Couple communication and romance

Romance is enhanced and deepened through respectfully and intimate communication and allows couples to share personal emotions or feelings with each other. Participants mentioned factors that enabled and accelerated their motivation to engage in sex with their husbands. These factors included strong emotional intimacy, effective communication skills, and true and caring companionship. Study participants pointed to the importance of deep emotional intimacy, romantic and friendly relationships, spending time together and enjoying each other in enhancing and maintaining women’s sexual motivation. Participant 18 related the following:

*“We have a romantic relationship with each other …it means we love each other a lot, we talk about our feelings easily, we often are together, especially on weekends and holidays. We enjoy being with each other and walking, laughing, hugging…We do not tolerate each other’s discomfort.”* (*post menopause*).

Husbands’ involvement in solving life and marital problems as well as their cooperation in housework were mentioned as contributing to women’s sexual motivation. Participant17 talked about her husband’s companionship as follow:

*“My husband is my best friend; he helps me a lot. For example we go shopping with each other; he helps me in housekeeping, we usually go on a trip every weekend, he participates in solving our life problems, in brief, we often are with each other. These [factors] motivate me to enjoy being sexually active.”* (post menopause).

Good and respectful communication skills, which can improve couples’ physical and emotional intimacy and strengthen mutual understanding, were said to facilitate and enrich spousal relationships and thus motivate women sexually. Participant 19 stated in this regard:

*“My husband can communicate with everyone so his communication is also excellent with me; he always holds me dear. We try to understand each other, especially if there are problems, by spending time and listening to each other. We mutually respect each other through honorable talking and behavior.”* (post menopause).

### Socio-cultural scripts

In addition to interpersonal and individual-level factors, social and cultural norms influence the way in which couples interact with each other. One of the main categories that emerged was sociocultural scripts, which comprises social, cultural and religious factors. Participants identify these scripts as reasons for diminished sexual motivation. Within this broad category, two sub­categories were extracted from the transcripts: sexual scripts and parental responsibility.

### Sexual scripts

Participants identified sexual scripts as the most common and most important factor that decreases their sexual motivation and causes them to withdraw from sexual behavior. Sexual scripts arise from widely shared gender- and culture-specific norms for sexual behavior. Iranian sexual scripts often have a strong hold over Iranian menopausal women, many of whom feel obligated to engage in sexual activities even when they do so unwillingly. The category of sexual scripts was further divided into the following subcategories: women’s obedience in sexual relationships, preserving marital life and fulfilling husband’s sexual needs.

Some women reported that they felt they were obligated to tolerate sex even if they did so unwillingly. They noted that they did so due to their commitment to be obedience in their sexual relationship, to preserve their marriage or to fulfill the husband’s sexual needs. They explained that these commitments or felt obligations negatively affected their sexual motivation. As participant 9 stated,

*“If I were not under some commitments, I wouldn’t lose my sexual motivations..... I bear difficult situations [in my] sexual life because of social and religious commitments…We are Muslim, we must obey holy Quran’s orders …it recommendeds fulfilling religious commitments, especially about obedience in sexual relationship.” (perimenopause).*

Participant 13 commented on the need to preserve marital life:

*“My life is important, life isn’t just sexuality. I’m socially obliged to stay with my husband….in my family divorce is ominous … ….Despite the loss of my sexual motivation, I tolerate harsh sexual life conditions to preserve my marital life after 30 years of marriage….I can’t lose my life at middle age*,” (perimenopause).

Participant 20 described fulfilling her husband’s sexual needs as follows:

*“It is both socially and religiously recommended to women to satisfy their husband especially due to their sexual needs…. Despite my reluctance regarding sex relations, I obliged myself to respond and fulfill my husband’s sexual needs to satisfy him.” (Age54, post menopause).*

### Parental responsibilities

Respondents indicated that responsibility for their children was another sociocultural barrier that had negative effects on their sexual motivations. Participants stated that Iranians tend to give priority to providing for, and responding to, their children’s needs over their own needs, including even their sexual desires. As participant 8 said:

*“Recently I couldn’t think about sex… my son is about 27 years old and he didn’t find a job …he is still single ….how can we enjoy sex?…He is young, his sexual need is more than us …my daughter has recently divorced and now, she lives with us…while she is crying every night …how can I think about having sex?”* (perimenopause).

Participant 5 expressed it thusly:

*“We have two young adolescent children, we are ashamed to have sex more, even about having physical closeness … I always wear covered clothes without any make up, especially in front of my son…you know… these erotic stimuli can motivate women. In this situation [without such stimuli] my sexual motivation decreases gradually.”* (post menopause).

### Sense of youthfulness

The final main category that arose from the transcripts was the sense of youthfulness, from which two sub-categories were extracted: having an active and happy life and keeping physically fit and attractive.

### Having an active and happy life

The participants identified experiencing an active and happy life as a key factor in facilitating sexual motivation. As participant 15 stated:

*“I’m sexually active and happy in comparison to most of our peers and friends because I planned for a happy life …especially about my leisure time …I go swimming with my friend… I take part in parties…. I plan happy weekends with my family.”* (post menopause).

### Maintaining physically attractiveness

Women’s attractive appearance was other factor that women mentioned as motivating and stimulating engaging in sexual behaviors. For example, participant 16 said:

*“When I care about my appearance, for example when I use make up, perfume and revealing, beautiful clothes that boost my attractiveness, my fantasies about sex usually increase.” (post menopause).*

## Discussion

This study explored the meanings generated through the lived experiences of Iranian women during their menopausal transition, some of which may challenge preconceived notions regarding sexuality and related motives. Overall, the women’s narratives about their sexual experiences tended to focus more on factors that inhibited, rather than on those that enabled, sexual relations.

While a previous study explored problem-solving strategies among Iranian women in the menopausal transition [4], to date, no study has explored the meanings Iranian women assign to sexuality during that life stage and the ways in which they process those experiences. The current study identified an array of sociocultural scripts that create the context in which peri-menopausal women experience and process sexual motivations, thus expanding and deepening our understanding of Iranian women’s experiences with respect to “intimate coupling” during this transitional period. Another major finding of this study was that women’s sexual lives during the menopausal transition are heterogeneous and varied, which is consistent with the findings of other research, including Hinchliff et al., a study conducted among British menopausal women [13]. We found that spousal relationships can be positive and elicit pleasant reminiscing and intimacy or, in other cases, can generate negative thoughts and ruminations, particularly among women who are emotionally disinclined to engage in sexual contact, thus confirming findings from a previous studies [23].

Participants in this study also indicated that relational factors, including intimacy and satisfaction can shape sexual experiences, which is consistent with previous research [24].

Not all participants experienced reduced or diminished sexual desire and motivation; participants explained that this was a result of romantic and rewarding intimate relationships. Enjoying strong emotional intimacy motivates spouses to engage in sex because they enjoy the feeling of being emotionally and physically close to their sexual partner, a finding similar to Beck et al. [17].Moreover, spousal intimacy is often a result of other aspects of the relationship between the partners [25]. In particular, it was reported that communication is an important factor; it can facilitate or impede intimacy in romantic relationships [25]. Research has shown that positive sexual experiences increase cognitive and emotional responsiveness [26], a finding that was supported by this study. Partners’ positive communication experiences are generally associated with high levels of intimacy and satisfaction [27]. Numerous studies indicated that joyful and intimate couples tend to enjoy more sexual intimacy and consequently become even happier and more satisfied [27, 28].

Conversely, couples in problematic relationships are likely to have unsatisfying sexual relations and, concomitantly, experience even greater relationship dissatisfaction [23]. As a result, dissatisfied and unhappy spouses are less sexually motivated and tend to engage in less sexual and physical contact [23].

Women’s commitment to socio-cultural scripts was one of the themes that emerged in this study as a factor that decreased sexual motivation in women. Some common Iranian sexual scripts, including those that obligate women, including menopausal women, to engage in sexual activities even when they are uninterested and unwilling, are based on inaccurate and misleading interpretations of religious texts. The reason for choosing this passive approach by many, perhaps the majority, of women is deeply rooted in cultural and traditional beliefs according to researchers who argue that sexual behavior is associated with gender socialization [4, 29].

In Iran women’s sexuality is strongly influenced by androcentric or patriarchal notions [15]. Women are culturally conditioned to please their husbands and fulfill their duties as a wife [15]. While the majority of Iranian women reported that their husbands are typically not concerned with women’s pleasure in sexual encounters, some reported that in the context of mutual love their husbands are particularly attentive to the wife’s pleasure during sex [30]. Hence, while few women reported openly expressing their sexual needs and desires, some mentioned that they tried to shift their strategy from one of silence to negotiation, seeking help, or sexual adjustment [31]. Notably, women sought care for sexual matters from health clinics only in the context of the reproductive life course and not in terms of enhanced sexual pleasure or practice [1].

While the majority of Iranians are Muslim, the fact that Islam respects women and portrays women as a center of kindness [32] has not necessarily translated into respectful treatment of women, as the study participants noted. According to Islamic tenets, a married man is obliged to treat his wife in a way to fulfill her desires for love and affection, including sexual relations [33]. Yet, as described above, it is not only men but also some women who have misinterpreted or misunderstood religious texts. Instead, the findings from this study suggest that cultural traditions and scripts dominate over religious precepts among many Iranians, which helps explain why some Iranian women believe they are obliged to acquiesce to their husbands’ sexual demands. This is consistent with the findings of a study by Bahri et al., which reported that Muslim menopausal women reported forcing themselves to meet the sexual demands of their husband [4].

The findings suggest that, in accordance with traditional Iranian sexual scripts, many men have neglected their duty while expecting that women will comply with their husbands’ expectations, even in the absence of intimacy and companionship. The findings suggest further that many women have also accepted a culture of obedience in sexual relationships, even when reluctant to do so. These findings are consistent with other studies conducted among British and Iranian menopausal women [4, 13], which found that participants who didn’t have pleasurable sex and were dissatisfied with such relationships, even to the extent of describing sex as abusive at some level, tended to respond by letting husband get on with sex or faking orgasms to facilitate a quicker ending.

Women who were dissatisfied with their relationships and described sex as coercive, complained of subjective pain in sexual intercourse. Lack of or inadequate lubrication due to the absence of sexual motivation and desire could be the main reason for such pain. Another possibility is the presence of genito-urinary syndrome of menopause that leads to pain. Women’s shame sometimes rendered them reluctant to seek help from healthcare providers for treatment [31]. A recent qualitative study of Iranian post-menopausal women (2017) revealed that despite the negative impact of vulvovaginal atrophy on women’s marriage and relationships, none of the participants reported ongoing treatment of their vulvovaginal atrophy with local low-dose estrogen [34].

In the Iranian culture, preserving marital life is important and vital for most women, many of whom believe that divorce during the menopausal transition and after a lengthy marital life is not compatible with social norms. Studies have confirmed that divorce, while legal, is difficult in Iran, particularly for women, and is considered socially inconvenient, if not unacceptable [35, 36].

While the narratives did reveal a range of negative forces on sexuality during the menopausal transition, there were also reports of factors that motived and enabled sexual relations during this time. Our findings support the results of studies that found women who were engaged in physical and social activities, including club membership, were more likely to be fit, happy and experience a slower aging process [37, 38] as well as more likely to enjoy improved sexual desire and motivation [38].

Attractiveness and physical appearance is also an important element of marital life, both in the West and in the East [39]. In a phenomenological study conducted in Iran, women described femininity as associated with youth; thus menopause, from their perspective, signaled the end of sexual relationships [3]. Likewise, women in the current study reported that maintaining a spirit of youthfulness and paying attention to beauty and physical appearance helped them maintain sexual relations, thus lending support to the long-standing assumption that physical attractiveness in a mate is more important for men than for women. This assumption has recently been put to the test by Meltzer et al. 2014, who found support for it in long-term, but not in short-term, relationships [39]. Women who were objectively found to be attractive at baseline experienced lower levels of decrease in marital satisfaction over a four-year period than was true of less attractive women [39].

Our study found that diminished sexual capacity, which was attributed to biological factors, had a dampening effect on participants’ motivation to engage in sexual behaviors. Desire discrepancy also contributed to decreased sexual motivation among the women in the study. Consistent with current research, it seems that some women in committed relationships feel they can meet their husbands’ sexual needs or demands, which leads to a sharp decline in, or end to, their sexual activity [4, 13]. Nevertheless, most of the women expressed concerns about their husbands’ unmet sexual needs. As a result, they considered themselves responsible for fulfilling their husband’s sexual needs [13], which this study suggests is related to sociocultural commitments. One study of sexuality at menopause found women are ‘responsible’ for their partner’s sexual needs due to gender differences in Chinese culture [5].One commonly reported reaction was to fake a sexual response, especially orgasms, to facilitate a quicker ending. They didn’t want to communicate their own distress to their husbands about their loss of sexual desire; instead, they sought to respond to their husband’s sexual needs and preserve the emotional intimacy of marital life. Some women worried about their husband’s possible betrayal/infidelity or that their husband would become more distant and isolated if they were to disclose their sexual problems to husband.

Previous research has identified the male partner’s sexual problems as a key reason that women with heterosexual partners discontinue sexual activity at midlife [13].This is consistent with the findings presented here; as reported above, some of the women complained of their husbands’ detachment from marital intimacy. They asserted that the relational context is more important than sex and their distress was mitigated when they enjoyed an intimate relationship with their husbands, whether with or without sex. The reason for men’s isolation is probably twofold. First, men may value sex for physical pleasure rather than relational intimacy and, secondly, research suggests that many men achieve relational intimacy through sexual intercourse [17].

Women’s reports cannot serve as a proxy for their husbands, nor can they give a full picture of the couple regardless of the topic. When it comes to sexual relations, the role of the husband is vital. Therefore, we strongly suggest that researchers design future studies to include interviews with husbands to reach new insights. As of this writing, the health care system does not provide medical support or socio-psychological guidance to midlife couples who are experiencing sexual problems during the menopausal transition. It is important to provide sexual health education and counseling, to encourage critical discussions regarding current sociocultural scripts and to create an environment that would enable men and women alike to adopt a healthy and happy lifestyle.

In Iran, a Muslim country, discussing sexual issues outside marriage is banned modesty and shyness related to this topic prevent couples from conversing about sex [39]; thus, it is difficult for couples to seek help. In the Iranian culture, it seems sexuality is influenced by patriarchal notions [30]. For example, the majority of men may not be aware of women’s pleasure or fulfillment in sexual relation. More likely neither they have learned about woman’s sexual needs nor help his wife to experience pleasurable orgasm. This poses the assumption that many of men consider sexual problems as their wives’ problem [30]. Thus, the tendency not to participate in negotiating sexual issues might have been the main factor that might prevent husbands from attending counseling or educational sessions [40]. To overcome to this barrier, the first possible step can be strategic planning for engaging men and boys in sexuality and reproductive health education; step two assess men’s knowledge and attitudes related to sexual and reproductive health; step three, to determine means by which gender norms affect men’s sexual performance; and finally identify and implement sexual and reproductive approaches to promote midlife’s’ sexual and reproductive health status.

There were several limitations in the present study. Participants were purposively selected, so the results can be generalized neither to Iranian women in general nor to Azeri-speaking women in Iran. This idiographic study, however, was not designed to generalize based on preconceived notions but to expand our understanding of the respondents’ experiences during the menopausal transition by eliciting women’s descriptions of those experiences in their own words. This allowed women to speak about their experiences at length without restricting their responses to predetermined categories.

Other limitations included the fact that talking about sexual issues in this community is difficult due to socio-cultural scripts; several appointments were required for interviews with some women due to their workload; and identifying appropriate locales for interviews sometimes entailed moving from one locale to another. More research is needed to explore both women’s and men’s sexual experiences to better understand and respond effectively to their relational and sexual needs during the menopausal transition.

## Conclusions

By limiting sexuality to the reproductive period of life, the women in this study did not focus on sexual wellbeing in the broader sense; instead, they regarded themselves as sexually healthy when no gynecological issues were experienced. The results of the present study could inform the design and implementation of interventions to promote women’s sexual health, particularly by eliminating barriers and preserving and enhancing motivational factors associated with sexuality.

## Abbreviation

FSD: Female sexual dysfunction
